# Investigating Tungsten Sulfide as a Counter Electrode Material in Dye-Sensitized Solar Cells

**DOI:** 10.3390/nano12162761

**Published:** 2022-08-12

**Authors:** Saket Chand Mathur, Soheil Rashidi, Wei Wei

**Affiliations:** Department of Mechanical Engineering, Wichita State University, 1845 Fairmount St, Wichita, KS 67260, USA

**Keywords:** dye-sensitized solar cells (DSSCs), tungsten sulfide, transition metal dichalcogenides (TMDs), counter electrodes, activated carbon

## Abstract

With the recent interest in renewable energy sources, dye-sensitized solar cells (DSSCs) have received a great deal of attention as a cheaper and more sustainable alternative to silicon-based solar cells. In a DSSC, the counter electrode performs the catalytic reduction of the electrolyte and electron collection. To perform this function adequately, platinum is the preferred material currently. To reduce the dependence of the DSSC on such an expensive material, alternatives such as activated carbon (AC) and two-dimensional transition metal dichalcogenides, and more specifically, tungsten sulfide (WS_2_), were considered. AC has shown great potential as a material for counter electrodes, whereas WS_2_ has unique physiochemical properties which warrant its exploration as an energy material. In this article, we synthesized and evaluated the performance of DSSCs with AC, WS_2_, and AC/WS_2_ composite counter electrodes. It was demonstrated that the performance of the WS_2_/AC composite counter electrode with a 1:2 ratio of WS_2_ to AC shows the highest performance with an efficiency of 6.25%.

## 1. Introduction

The global demand for energy over the years poses a significant challenge to future generations if there is no diversification of energy sources [1]. Energy demand is dramatically increasing while there is a lack of energy resources to replace fossil fuels, and there is already a reduction in oil assets extant on the crust of the earth [2]. The continued use of these fossil fuels will lead to a negative impact on the environment and living species, and lead to the destruction of ecosystems, impacting the human population [3]. In order to address these problems, the scientific community has shown a great tendency to study renewable energy resources, which are compatible with today’s environmental policy. Solar energy has a large energy reservoir and easy availability, which puts it at the forefront of renewable energy sources [4]. Thus, to optimize energy management and improve environmental security, it is vital to utilize the solar spectrum to generate power through the application of photovoltaic devices, which are capable of producing electricity directly from sunlight without any moving mechanical components. Because of the technological maturity and high efficiency, silicon-based solar devices are enjoying a major share in the overall market [5,6]. Silicon-based solar devices have a complicated production process and require many rare earth metals for the doping of silicon. These two factors increase the cost of this technology for urban and other commercial applications, limiting their deployment [7].

The dye-sensitized solar cell (DSSC) is a third-generation solar cell, which is a promising alternative to conventional silicon solar cells in terms of optimum efficiency and ease of manufacture [8,9,10]. In addition to those advantages, they are lightweight, flexible, and have a low cost per unit [11]. The DSSC consists of five important components, i.e., the substrate (conductive substrate or conductive film-coated substrate), semiconductor nanostructure (photoanode), sensitizer (dye), electrolyte, and catalyst-coated counter electrode [12]. To measure the performance of a solar cell, the efficiency and the fill factor of the device are considered. The fill factor determines the maximum power of the cell with respect to the open-circuit voltage and the short-circuit current. To generate usable electrical energy from sunlight, the DSSC presents three steps: (1) photoexcitation of dyes by transferring an electron to the conduction band of the semiconductor (in this case TiO_2_); (2) regeneration of the oxidized dye molecules with electron donation from the redox couple present in the electrolyte; and (3) migration of the electron through the external load to complete the circuit [13,14]. The entire operation takes place with the help of all the components of the DSSC [15,16]. The collection of electrons from the external load is performed by the counter electrode (CE), which also catalyzes the reduction of I_3_^−^ to I^−^, thereby realizing the regeneration of the sensitizer. The most commonly used CE is platinum (Pt), which is deposited on the fluorine-doped tin oxide (FTO) conductive glass. However, the high cost of Pt has reduced the commercialization of DSSCs. Transition metal dichalcogenides (TMDs) have been established to have good electrical and thermal conductivity, along with the durability of the covalent bond and high melting temperature. The investigation of graphene-like layered metal dichalcogenides is still in its early stages. Tungsten dichalcogenides, which are the same as other TMDs, offer active sites for catalytic reactions [17,18]. Based on recent studies, the performance of conventional Pt counter electrodes in DSSC devices can be surpassed when Pt is replaced by TMDs [9,19]. Replacing platinum counter electrodes to develop low-cost, catalytically active, and stable counter electrodes by utilizing WS_2_ is one of the hot topics among researchers nowadays [20,21,22,23]. In addition to TMDs, carbon-based materials are proven to increase efficiency and reduce the cost of DSSCs [10].

To use WS_2_ as a counter electrode in DSSCs, thin films of the material are usually prepared and layered on a conductive FTO glass. This can be achieved using doctor blading as demonstrated by Li et al. [24]. The thin films of WS_2_ show promising results, but suffer in comparison to other materials due to relatively worse conductivity [25]. To counter this, Wang et al. made a multilayer CE with a layer of activated carbon (AC) on top of the WS_2_ layer [26]. Although they could not observe a chemical bonding between carbon and WS_2_ nanoparticles after the coating process, the presence of the amorphous layer of carbon increases the conductivity between WS_2_ particles. The power conversion efficiency (PCE) of the carbon-coated WS_2_ counter electrode was 5.5%, which is comparable to that of the Pt counter electrode (5.6%). A novel WS_2_/carbon film was developed by Shen et al. for use as a counter electrode after the sulfurization of a mesoporous WO_x_/carbon film [27]. The continuous carbon layer provides a facile electron transfer and electrolyte diffusion to the 3D WS_2_ framework, contributing high electrocatalytic activity and fast reaction kinetics for the redox of I_3_^−^/I^−^. The power conversion rate of 7.71% was reported for this WO_x_@WS_2_@carbon composite counter electrode, which is favorably comparable to that of the WO_x_/carbon CE (6.00%) and conventional Pt counter electrode (7.34%). Finally, it was reported that the PCEs were 7.25% and 4.67% for WO_2_ CE and WO_3_ CE, respectively [28]. It is theorized that the carrier concentration increases from WO_3_ to WO_2_ as the coordination number of the anion decreases, leading to a higher charge transfer rate [29]. These findings allow us to assume there is an optimum amount of carbon that would enhance the performance of the CE to the highest levels possible in this device. However, care must be taken as a rich carbon coating can cover the active sites of WS_2_, which decreases the overall bonding strength.

In this report, we synthesized the WS_2_/activated carbon composites as counter electrode materials for DSSCs and explored the impact of the composition of the composite materials.

## 2. Experimental Methods

### 2.1. Preparation of Photoelectrode

Titanium oxide (TiO_2_) powder was purchased from Sigma-Aldrich (Sigma Aldrich inc., St. Louis, MO, USA) and used as received. The photoelectrodes were prepared by mixing 0.1 g of TiO_2_ powder in 1 mL of ethanol. Then, the paste was applied to a fluorine-doped tin oxide (FTO) surface by the doctor-blading method to prepare films with a dimension of 0.5 cm by 0.5 cm, leading to an overall surface of 0.25 cm^2^. The process was followed by sintering at 500 °C for 40 min. After the samples cooled down, a dye bath was prepared by mixing 0.0042 g of N719 in 20 mL of ethanol, and photoelectrodes were placed into the dye for 72 h. Powder X-ray diffraction measurements of electrode materials were performed to determine their crystal structure by using a Rigaku MiniFlex II X-ray diffractometer (Rigaku, Austin, TX, USA).

### 2.2. Preparation of Counter Electrode

The tungsten sulfide (WS_2_) was purchased from Alfa Aesar (Alfa Aesar, Tewksbury, MA, USA) and used without any chemical modification. To establish the performance of the materials, a batch of counter electrodes was made with pure WS_2_. To prepare the thin film, 0.6 g of WS_2_ was suspended in 1 mL of ethanol to form a paste for uniform distribution. The resultant paste of this mixture was hand-ground for 30 min to achieve a finer and more uniform paste. The paste was doctor-bladed on the FTO glass substrate. The film was then placed in an oven at 65 °C for 2 h to eliminate the moisture. The fabrication of the WS_2_/AC CEs was the same as described above by varying ratios of AC vs. WS_2_. Platinized CEs were fabricated by dropping a platinum precursor (chloroplatinic acid hexahydrate) on a cleaned FTO glass substrate and sintering it at 500 °C for 40 min. A scanning electron microscope (Phenom Pharos system, Nanoscience Instruments, Phoenix, AZ, USA) was employed to characterize the morphologies of electrode materials. This device uses a Schottky field emitter as the source and has a maximum magnification of 1,000,000×. The resolution of the equipment was 2.5 nm at 15 kV (SED), 4 nm at 15 kV (BSD), and 10 nm at 3 kV.

### 2.3. Preparation of Electrolyte

The electrolyte for the DSSC was made by mixing 0.100 g of I_2_, 0.034 g of LiI, 0.318 g of tert-butylpyridine, 1.600 g of 1-buty-3-methylimidazolium, and 0.600 g of guanidine thiocyanate into 10 mL of solvent: 8.5 mL of ethanol and 1.5 mL of valeronitrile. The electrolyte for CV measurement was made by adding 0.134 g of LiI to 1.066 g of LiClO_4_ and 0.027 g of I_2_ and dissolving the mixture in 100 mL of acetonitrile. All materials for the electrolytes were purchased from Sigma-Aldrich (Sigma Aldrich Inc., St. Louis, MO, USA) and used as received. 

### 2.4. Fabrication and Evaluation of DSSCs

The DSSCs were fabricated by layering the dye-immersed TiO_2_ photoelectrodes facing the active area of the counter electrodes. The prepared electrolyte solution was filled in between the electrodes before they were attached. The I-V characterization was performed with the Gamry Interface 5000e (Gamry Instruments, Warminster, PA, USA) equipment. The scanning rate was 10 mV s^−1^ with a step size of 10 mV. The ORIEL LED Solar Simulator was used to simulate sunlight at an intensity of 100 mW cm^−2^. For the cyclic voltammetry measurement, the three-electrode method along with the Gamry Interface 5000e (Gamry Instruments, Warminster, PA, USA) equipment was used. The three electrodes consisted of a reference electrode of Ag/AgCl, a counter electrode of Pt, and the working electrode, which is the electrode under investigation. The scan rate of 20 mV s^−1^ was used with a step size of 20 mV. The electrochemical impedance spectroscopy (EIS) characterization was carried out with the Gamry Interface 5000e (Gamry Instruments, Warminster, PA, USA) with scanning frequencies between 100 kHz and 10 MHz at open-circuit voltage.

## 3. Results and Discussions

**Figure 1:**
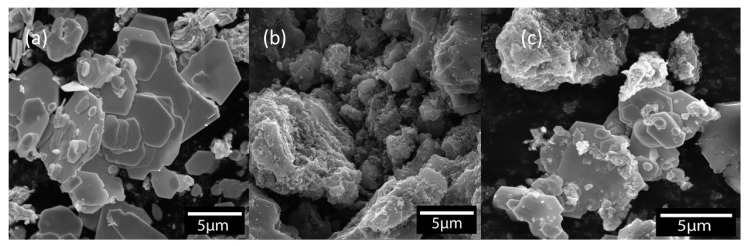
SEM images of (**a**) pure WS_2_, (**b**) AC, and (**c**) WS_2_-AC (1:2) composite.

In order to investigate the morphologies of counter electrodes in DSSCs, the scanning electron microscope (SEM) was employed and the obtained SEM images are shown in Figure 1. The WS_2_ flakes can be observed in Figure 1a; they are very thin but in all different sizes. The surface of the WS_2_ flakes looks very smooth in most areas. On the other hand, the AC has a very porous structure while the particles are bulkier in comparison with WS_2_, as shown in Figure 1b. Differences in the structures of these two powders must lead to an interesting structure. Figure 1c illustrates the SEM image of WS_2_/AC composites with a ratio of 1:2. The smaller particles of AC are placed on the surface of the WS_2_ flakes and, therefore, increase the free space in the structure. Based on the SEM observations, it is notable that mixing WS_2_ with AC was successfully placed and good uniformity of the structure is detected in various areas of the composite.

In the literature, the porosity of AC and WS_2_ was reported as 720 m^2^/g and 3.49 m^2^/g, respectively. Thus, the total porosity of WS_2_/AC (1:2) is expected to be more than that of AC and WS_2_ separately. Therefore, theoretically, a higher surface area should be accessible. Hence, the catalytic activity of the composite is expected to be higher than that of both WS_2_ and AC. Another advantage of the porous structure of the composite is that there would be a better surface connection between the counter electrode and the electrolyte. In other words, a better connection on the surface between the CE and electrolyte means a better charge transfer ability for electrons. Consequently, the charge transfer resistance of the WS_2_/AC (1:2) composite is expected to be lower than that of AC, which is later confirmed by the electrochemical impedance spectrum (EIS) results.

**Figure 2:**
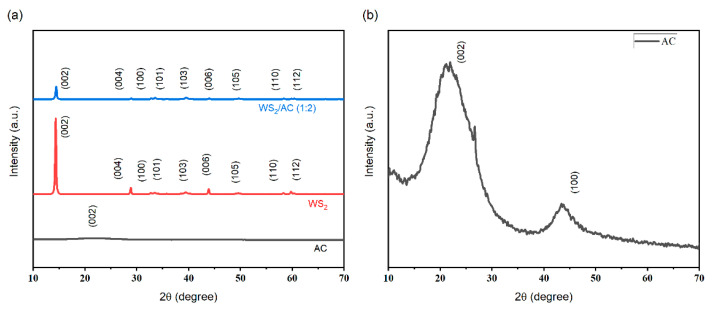
XRD patterns of (**a**) the synthesized WS_2_, AC, and WS_2_/AC (1:2) counter electrodes used in DSSC and (**b**) pure activated carbon.

The crystal structures of electrode materials were further evaluated by X-ray powder diffraction (XRD) using the Rigaku MiniFlex II X-ray diffractometer. The comparison between the XRD results of WS_2_, AC, and WS_2_/AC (1:2) is illustrated in Figure 2. Since the highest recorded peak intensity of WS_2_ is much larger than that of AC, the correlated peak of AC is not significant. The peak intensity of the WS_2_/AC composition decreases in comparison with the peaks of pure WS_2_. The reason is that the AC powder has a much smaller particle size in comparison with the WS powder. Hence, the composition has a smaller particle size in comparison to that of pure WS_2_. The average particle sizes of the WS_2_, activated carbon, and composite mixture are found to be 29.68 nm, 0.96 nm, and 22.46 nm, respectively.

As shown in Figure 2, the highest intensity is observed at 14°, which is related to the (002) plane. The other two peaks with the highest intensity values could be found at 27° and 43° corresponding to the (004) and (006) planes, respectively. The XRD pattern of the AC has a very wide peak that is observed in a range from 16° to 30°. The maximum intensity of this peak is observed at 21° while another peak can be found at 43°. The XRD pattern of the WS_2_/AC (1:2) shows a similar pattern with pure WS_2_. However, due to the presence of AC, there is a wide and low-intensity peak from 16° to 30°. All other high-intensity peaks are attributed to the presence of WS_2_.

**Figure 3:**
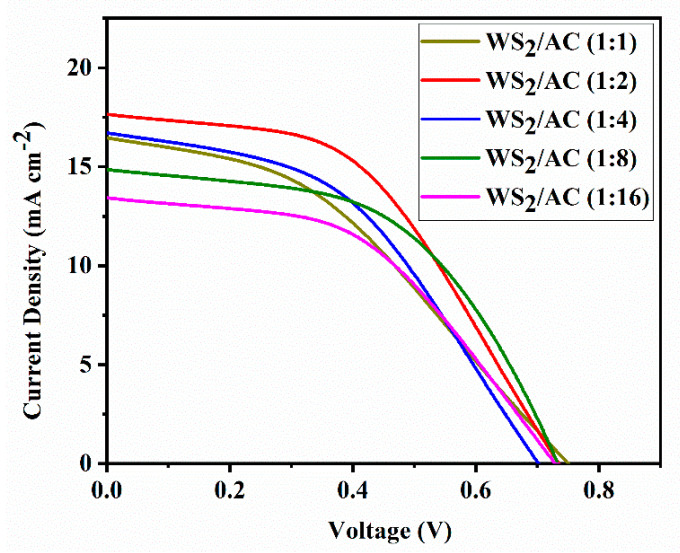
Current-voltage characteristics of the DSSCs assembled with different ratios of WS_2_/AC counter electrodes measuring under AM 1.5 solar simulator with 100 mW cm^-2^ illumination.

To investigate the performance of WS_2_/AC composite materials as counter electrodes in DSSCs, the I-V measurements were carried out. The results of I-V measurements for different ratios of WS_2_/AC composites are illustrated in Figure 3. The highest and the lowest short circuit current (I_SC_) are obtained at the ratio of 1:2 (17.65 mAcm^−2^) and 1:16 (13.11 mA cm^−2^), respectively. However, the open-circuit voltage (V_OC_) is almost the same for most of the DSSCs, ranging between 0.70 V to 0.75 V. Moreover, the WS_2_/AC composite with the ratio 1:2 has the most rectangular shape (higher fill factor) in comparison with the other ratios. In other words, the performance (6.25%) of composite material-based DSSCs would be the best when the ratio of 1:2 is used. Hence, we compared the result of WS_2_/AC (1:2) with WS_2_- and AC-based DSSCs to obtain a better understanding of performance improvement.

**Figure 4:**
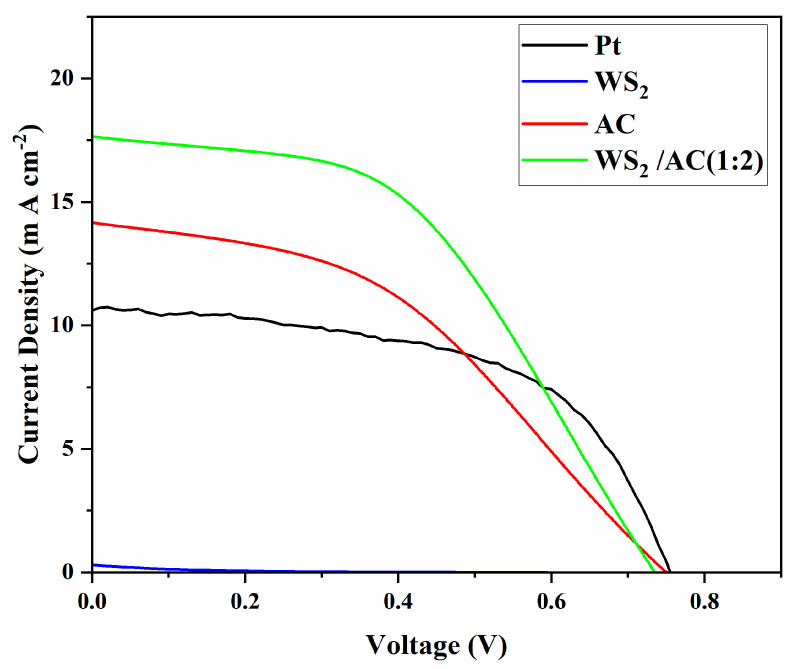
Current-voltage characteristics of the DSSCs assembled with different counter electrodes (WS_2_, AC, WS_2_/AC, and Pt), measured under AM 1.5 solar simulator with 100 mW cm^-2^ illumination.

Figure 4 shows the I-V curves of DSCCs with WS_2_, AC, and WS_2_/AC composites as counter electrodes. The WS_2_-based DSSC has very small values for both I_SC_ and V_OC_, suggesting that its performance is very poor. Although the AC-based DSSC shows a slightly higher V_OC_ than the WS_2_/AC (1:2) composite-based DSSC, there is a significant difference between the I_SC_ for these two different devices.

The parameters, such as FF and *η*, are then calculated from the graph. The parameters and photovoltaic performance of the prepared cells are shown in Table 1. However, the *η* of a DSSC does not comprise the amount of light captivated by the dye but measures the overall conversion of applied light to electrical power. The *η* of the DSSC was obtained by calculating the ratio of the maximum power generated by the device to the intensity of the incident light from the solar simulator on the active area of the device. The WS_2_/AC composite cell with the ratio 1:2 achieved the best efficiency, as high as 6.25%, which is significantly higher than that of WS_2_- and AC-based cells at 1.79% and 4.50%, respectively. It is of great importance that all the cells made of the WS_2_/AC composite have shown a better performance than both WS_2_- and AC-based cells. In addition, the FF values are in a range between 0.39 and 0.52. Furthermore, the Pt-based DSSC was also fabricated as a comparison. As shown in Table 1, the WS^2^/AC-based DSSC shows higher efficiency (6.25%) than that of the Pt-based device (4.5%).

Based on a simple comparison of the open-circuit voltage amount of WS_2_ samples and WS_2_/AC (1:2) samples from Table 1, we found a decrease in the V_OC_ for CEs made of the WS_2_/AC composite. Although the V_OC_ corresponds to the energy difference between the Fermi level of the semiconductor and the redox energy level of the redox couple theoretically, the decrease in the value of the V_OC_ is due to the over potential at the counter electrode in practice [30]. The increase in the amount of AC composition of the CE could explain the change in V_OC_ values.

It is of great importance to mention that the short-circuit current is identical to the light-generated current. In other words, I_SC_ is similar to the generation and collection of light-generated carriers, and therefore, it is the largest current that could be drawn from the DSSC. There are several parameters that could have an influence on the I_SC_ including the area of the solar cell, the number of photons (known as the power of the incident light source), the optical properties, and the collection probability of the solar cell. The surface area and the incident light power are the same for all the DSSCs and could not play a role in the observed I_SC_ difference. Hence, the difference in I_SC_ is believed to be caused by a difference in the diffusion length that could be varied based on the absorption and reflection of the solar cell, as well as the surface passivation.

**Figure 5:**
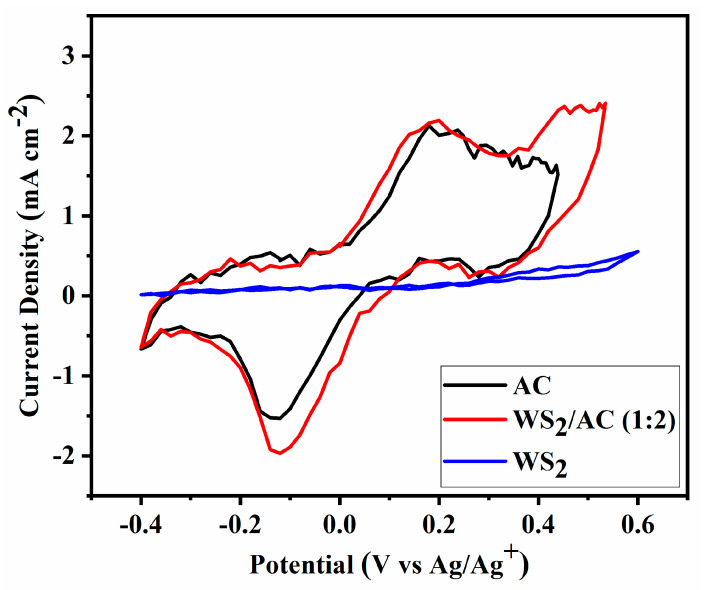
The cyclic voltammetry of the DSSCs is assembled with different counter electrodes (WS_2_, AC, and WS_2_/AC).

Cyclic voltammetry was used to examine the active surface area of the prepared cells. The reduction–oxidation abilities of the WS_2_-, AC-, and WS_2_/AC-based CEs on the I^−^/I_3_^−^ redox couple were studied at a scan rate of 20 mV s^−1^ and step size of 20 mV at room temperature. The CV measurements were performed between −0.4 and 0.6 V using a three-electrode configuration. As the CV results can be seen in Figure 5 the WS_2_ curve indicates an almost linear behavior where the very low area of the loop suggests that the electric property of this cell is very poor. Both AC and WS_2_/AC cells show a much higher loop surface compared with WS_2_; however, the WS_2_/AC composite cell has a smaller peak-to-peak separation (E_pp_ = 0.32 V). Although the E_pp_ of the composite CE is almost equal to that of the AC, the composite CE has a current density of 2.19 mA cm^−2^ which is higher than that of WS_2_- and AC-based cells. The obtained results illustrate that the WS_2_/AC composite can increase the catalytic activity of the WS_2_- and AC-based DSSCs as theorized when looking at the SEM imaging.

To have a better understanding of the electrical behavior of the cells, such as charge transport, transfer, and accumulation processes in the cell, the EIS measurement was used via the two-electrode method. A typical EIS spectrum of a DSSC illustrates information about charge transport as a result of electron diffusion through the photoelectrode (TiO_2_) and ionic diffusion in the electrolyte solution. In addition, the EIS spectrum indicates charge transfer attributable to the electron back reaction at the FTO/electrolyte interface, recombination at the TiO_2_/electrolyte interface, and regeneration of the redox species at CE/electrolyte interfaces.

**Figure 6:**
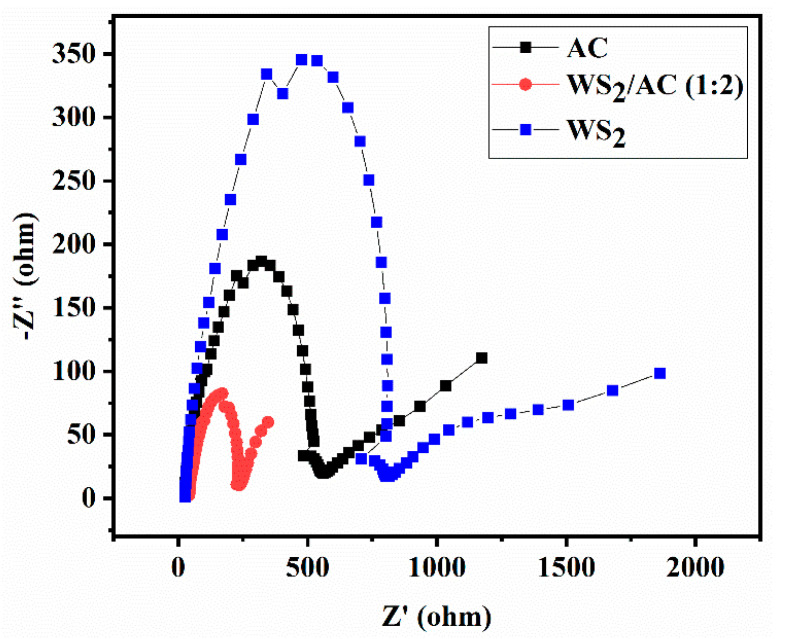
Nyquist plot of the DSSCs assembled with different counter electrodes (WS_2_, AC, and WS_2_/AC).

The Nyquist plot of the WS_2_, AC, and WS_2_/AC (1:2) cells at different potentials is shown in Figure 6. As can be seen, the charge transfer resistance at the interface between the CE and electrolyte is much lower for the WS_2_/AC composite cell in comparison with AC and WS_2_ cells. Although the second minimum point is not observed for any of the cells, by extrapolating the plots, it can be explained that the charge transfer resistance between the photoelectrode and electrolyte shows a similar change seen in the CE/electrolyte interface, which indicates that the addition of the WS_2_ reduces the resistance between the interface.

## 4. Conclusions

The use of tungsten dichalcogenides, and more specifically, that of WS_2_ with activated carbon, as a CE material shows better catalytic activity due to increased porosity, leading to a better performance compared to pure WS_2_. The WS_2_/AC composite counter electrode indicates a smaller peak-to-peak gap and a higher current density than that of WS_2_ or AC CEs, proving this composite CE has a better catalytic activity in comparison with pure WS_2_- and pure AC CE-based DSSCs. Moreover, the charge transfer resistance in both interfaces between the CE/electrolyte and PE/electrolyte is much smaller than that of pure WS_2_- and pure AC CE-based DSSCs. This result from the EIS measurement illustrates the better conductivity of the WS_2_/AC composite CEs, leading to a better performance of DSSCs. The constructed composite DSSC with the ratio of 1:2 between WS_2_ and AC exhibited a high-power conversion efficiency of 6.25%, which is much higher than that of WS_2_ (1.79%) and AC (4.50%)-based DSSCs. This novel WS_2_/AC-based DSSC opens up opportunities for a variety of optoelectronic and photoelectrochemical applications.

## Figures and Tables

**Table 1:** Performance of various cells made with WS_2_, AC, and a combination of both with different ratios.

Materials	J_sc_ (mA cm^−2^)	V_oc_ (V)	FF	η (％)
**AC**	14.17	0.75	0.42	4.50
**WS_2_**	3.50	0.53	0.43	1.79
**WS_2_/AC (1:1)**	16.46	0.75	0.39	4.87
**WS_2_/AC (1:2)**	17.65	0.74	0.48	6.25
**WS_2_/AC (1:4)**	16.71	0.70	0.45	5.29
**WS_2_/AC (1:8)**	14.86	0.73	0.52	5.71
**WS_2_/AC (1:16)**	13.43	0.73	0.48	4.74
**Pt**	10.62	0.56	0.56	4.50

## Data Availability

Data can be available upon request from the authors.
